# Cherokee Medical Society

**Published:** 1889-03

**Authors:** 


### CHEROKEE MEDICAL SOCIETY.

The Cherokee County Medical Association held its fourth quarterly meeting at Rusk, Texas, January 1, 1889. Dr. McCord, the President, in the chair, and in absence of the Secretary, Dr. J. K. Frazier was elected Secretary pro tem. The attendance was less than usual owing to sickness, but nevertheless there was much interest manifested. After disposing of the minutes of last meeting, the report of the standing committee on collective investigation was called for, but on motion was postponed until after the reading of the essays.

The President read his quarterly address with such recommendations as he thought proper. His address was terse, practical, pointed and very interesting to the association. Prominent among his recommendations, (after the election of officers for the ensuing year,) was the appointment of a committee to confer and act with the State committee on medical legislation. On motion a committee consisting of Drs. J. N. B. Guinn, J. K. Sturdevant and W. G. Jameson, were appointed to consider the suggestions as set forth in the President's message. On motion a recess was taken until half past one.

At half past one the house was called to order, Dr. McCord in the chair. The committee on credentials reported the application and acceptance of Dr. C. Cannon. On motion the application was received.

The committee appointed to consider the suggestions made by the President in his message, reported favorably and argued the adoptiou of same. As a committee on medical legislation the chair appointed Dr. J. N. B. Guinn, of Alto, Guinn, of Rusk, Collins, Frazier, and on motion he was added also. Dr. J. N. B. Guinn read a very interesting paper on pneumonia, and as the committee on collective investigation had investigated the subject of pneumonia also, it was agreed that the committee report and have both papers discussed at once; in the mean time, Dr. Jameson made an exhaustive and interesting report for the committee. Dr. McCord also read a minority report, which was specially interesting to the association, taking high and firm grounds against the pestiferous little bugs, spoken of by Klebbs and others.

Drs. McCord, Guinn, of Alto, Guinn, of Rusk, Jameson and Smith, participated in the discussion of these papers. Dr. Frazier read an able paper on catarrhal fever, which evoked a lively discussion by Drs. McCord, Jameson and Guinn. A vote of thanks was passed for the writers of the papers and to the committee on collective investigation. On motion it was resolved that the President appoint a committee of three on collective investigation, one from each town and vicinity, and on motion the President was added, after which the election of officers for the ensuing year resulted as follows:

J. N. B. Guinn, President; First Vice President, Dr. Collins; Second Vice President, Dr. Spring; Third Vice President, Dr. Boston; Secretary, Dr. J. H. Evans; Assistant Secretary, Dr. W. G. Jameson.

The committee on appointment of essayist for next metting, reported as follows:

Dr. A. W. Boston, subject—Cholera-Infantum; Dr. J. H. Evant, subject—Puerperal Eclampsia; Dr. B. F. Brittain, subject—Empyema; Dr. J. F. Johnson, subject—Cramp; Dr. McCord made a beautiful retiring address, giving a concise and instructive history of our young association whose tendency for improvement both socially and professionally is onward and upward, and though the child is less than one year old, by the guidance of its God Father and his willing coadjutors it is walking. Dr.

Guinn in assuming his responsibilities as presiding officer, thanked the members in appropriate terms for the honor conferred, and appointed a committee on credentials, consisting of Drs. Wilson, Smith and Frank Fuller.

Minutes read and adopted.

On motion the meeting adjourned until the next regular meeting, which is to be held in Alto the first Thursday in April, 1889. J. K. FRAZIER, M. D., Secretary Pro tem.

For J. H. EVANS, M. D., Secretary.

---

THE AUSTIN DISTRICT MEDICAL SOCIETY will hold its sixth quarterly meeting in Austin, Texas, March 21st inst. The following papers will be read and discussed :

"Vis Medicatrix Naturæ, how far the physician should trust to it, etc," by J. W. Harrison, M. D., of Dripping Springs. The discussion will be opened by Dr. R. M. Swearingen. "A report of a case of Aneurism of the Abdominal Aorta, with Post Mortem," by Frank P. McLaughlin, M. D., of Austin ; discussion opened by Dr. W. P. Flemming, of Georgetown. "Carbolic Acid Poisoning, with Treatment," by W. T. Richmond, M. D., of Manor ; dicussion opened by Dr. Sam Cunningham, of Elgin. "Opium Poisoning, with Treatment," by W. A. Morris, M. D. discussion opened by Dr. H. L. Hardy, of Paige. "Mastoid Abscesses," by T. J. Tyner, M. D., of Austin ; discussion opened by Dr. W. H. Way, of Austin. "Insanity Continued, Special or Immediate Causes," by A. N. Denton, M. D., of Austin ; discussion opened by Dr. John Preston, of Lunatic Asylum. "Foreign Bodies in the Air Passages," by G. W. Christian, M. D., of Burnet ; discussion opened by Dr. G. B. Underhill, of New Braunfels. Voluntary papers and reports of cases are always in order.

It will be observed from the above list of papers, that an interesting and attractive programme is in waiting for the meeting, and, therefore, it is safe to say a good time will be had. Every physician who is accessible to Austin should avail himself of the profits and pleasures of these meetings. There have never been less than thirty in attendance at any meeting, and the Society is now fifty-four strong and flourishing.

Dr. W. A. Morris is president, and Dr. T. J. Bennett, secretary.

---

PAPERS TO BE READ before the Section on Gynecology at the meeting of the Texas State Medical Association in San Antonio on April 23rd next, will now be appreciatively received by either of the undersigned.

No paper will be read at the meeting of the Section unless it or its title, with an epitome of its contents, has been in the hands of either the chairman or the secretary previous to that time.

B. E. HADRA, M. D.,
Chairman Section on Gynecology,
GEO. H. LEE, M. D., Sec'y. Galveston, Texas.

---

THE Alabama Medical Association will meet in Mobile April 9th prox.

